# Analysis of how a complex systems perspective is applied in studies on socioeconomic inequalities in health and health behaviour—a call for reporting guidelines

**DOI:** 10.1186/s12961-024-01248-x

**Published:** 2024-12-05

**Authors:** Andrea L. Mudd, Michèlle Bal, Sanne E. Verra, Maartje P. Poelman, Carlijn B. M. Kamphuis

**Affiliations:** 1 Department of Interdisciplinary Social Science- Social Policy and Public Health, Utrecht University, PO Box 80140, 3508 TC Utrecht, The Netherlands; 2 Chair Group Consumption and Healthy Lifestyles, Wageningen University & Research, Hollandseweg 1, 6706 KN Wageningen, The Netherlands

**Keywords:** Complex systems, Methodology, Reporting standards, Simulation models, Conceptual models, Socioeconomic inequalities, Health, Health behaviour

## Abstract

**Background:**

A complex systems perspective is gaining popularity in research on socioeconomic inequalities in health and health behaviour, though there may be a gap between its popularity and the way it is implemented. Building on our recent systematic scoping review, we aim to analyse the application of and reporting on complex systems methods in the literature on socioeconomic inequalities in health and health behaviour.

**Methods:**

Selected methods and results from the review are presented as a basis for in-depth critical reflection. A traffic light-based instrument was used to assess the extent to which eight key concepts of a complex systems perspective (e.g. feedback loops) were applied. Study characteristics related to the applied value of the models were also extracted, including the model evidence base, the depiction of the model structure, and which characteristics of model relationships (e.g. polarity) were reported on.

**Results:**

Studies that applied more key concepts of a complex systems perspective were also more likely to report the direction and polarity of relationships. The system paradigm, its deepest held beliefs, is seldom identified but may be key to recognize when designing interventions. A clear, complete depiction of the full model structure is also needed to convey the functioning of a complex system. We recommend that authors include these characteristics and level of detail in their reporting.

**Conclusions:**

Above all, we call for the development of reporting guidelines to increase the transparency and applied value of complex systems models on socioeconomic inequalities in health, health behaviour and beyond.

**Graphical Abstract:**

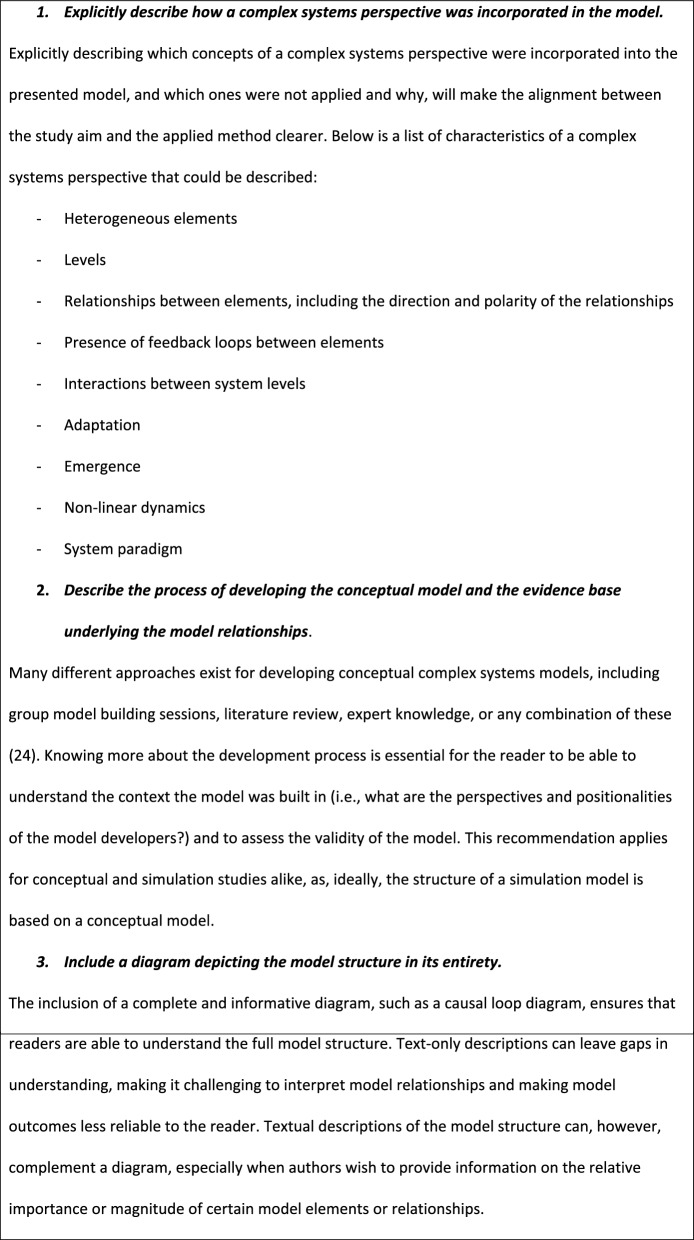

## Rationale

In the past 20 years, interest in applying a complex systems perspective to understanding the dynamics underlying socioeconomic inequalities in health and health behaviour has grown (e.g. [1–4]). A complex systems perspective combines systems theory and complexity science, in which health outcomes are considered to be an emergent property of the system as a whole [5]. Complex systems models include factors at multiple levels of influence and specify feedback loops between these factors. This sets complex systems models apart from most traditional approaches to studying socioeconomic inequalities in health, which often focus on linear relationships between single factors (e.g. quality of neighbourhood infrastructure) and lead to policy recommendations centring around single factors and outcomes (e.g. physical activity) [5]. For example, in an approach grounded in a complex systems perspective, the ways in which the quality of neighbourhood infrastructure is intertwined with other resources neighbourhood residents have access to (e.g. money, time or social support), prevailing cultural norms in the neighbourhood and how residents use the available infrastructure (for physical activity and other behaviours, e.g. socializing or consuming alcohol) may be viewed as just one part of what drives socioeconomic inequalities in health in that community. The parts or mechanisms in a complex system come together as more than the sum of their parts, in nonlinear and sometimes unexpected ways to influence the system’s behaviour (here, socioeconomic inequalities in health). Adopting a complex systems perspective may more effectively depict the complexity of real life processes, supporting public health policymakers and other stakeholders in meeting the challenges posed by systematic socioeconomic inequalities in health and health behaviour [6].

A complex systems perspective also introduces new analytical and conceptual challenges to understanding and modelling real-life processes, creating a potential gap between awareness about the usefulness of a complex systems approach and its implementation in research [7]. Taking stock of how existing studies on socioeconomic inequalities in health have applied a complex systems perspective may reveal opportunities for this important field of research to continue developing.

In our recent systematic scoping review [8], we provided an overview of 42 studies that modelled socioeconomic inequalities in health and health behaviour from a complex systems perspective using conceptual models, simulation models or both. Conceptual methods to modelling a complex system entail representing the system’s causal structure, and simulation methods entail formalizing and quantifying the system’s causal structure (e.g. agent-based models or systems dynamics models) [1]. The main focus of the systematic scoping review was to summarize and analyse the content of the identified models. In the content-focused review, we assessed the quality of included studies by evaluating the evidence each complex systems model was based on and the extent to which key concepts of a complex systems perspective were applied. During this quality assessment process, we found that key concepts of a complex systems approach were applied to varying degrees. During the review process, we also noticed that certain reporting styles aided our ability to understand the model structures in the studies we identified, while other reporting styles hindered our understanding. Clear reporting styles, in our view, are crucial for the interpretability and applied value of complex systems models, both for researchers and in practice (e.g. to inform the selection of policies). Given these preliminary observations, additional critical reflection on how complex systems methods have been applied on the subject of socioeconomic inequalities in health and health behaviour and how these methods have been reported on in publications is warranted [4]. This critical reflection on the application of and reporting on complex systems methods is the focus of this manuscript.

In this short manuscript, we aim to analyse how the studies identified in our systematic scoping review applied complex systems methodologies and how these studies reported on the methods applied. In this analysis, we [1] assess and critically discuss current applications of a complex systems perspective in the literature on socioeconomic inequalities in health and health behaviour and [2] provide recommendations for the development of comprehensive reporting guidelines, aimed at improving the transparency and applied value of future complex systems models. This short manuscript is based on data extracted in the process of conducting our systematic scoping review [8]. In order for this manuscript to function as a stand-alone piece, selected methods and results related to the application of a complex systems approach that are published elsewhere are briefly repeated here. What this manuscript adds to existing literature is an in-depth analysis and critical reflection on how complex systems methods were applied and reported on.

## Methods

The complete methodology for the systematic scoping review, which adhered to the Preferred Reporting Items for Systematic Reviews and Meta-Analyses (PRISMA) checklist, can be found in the main manuscript [8]. In short, we searched SCOPUS, Web of Science and PubMed from database start dates to April 2023 for studies that: [1] concern the adult general population in high-income countries belonging to the OECD [2, 9], contain an original or adapted conceptual or simulation model, [3], self-identify as having applied a complex systems perspective [4], include a measure of socioeconomic position and [5] include a health or health behaviour outcome relevant for the adult general population.

A traffic light-based instrument was used to assess the extent to which key concepts of a complex systems perspective were incorporated into the studies identified in our scoping review. Key concepts of a complex systems perspective were selected from prominent literature about complex systems in the context of socioeconomic inequalities in health [1, 10] along with literature about complex systems more generally [11, 12]. The format of our instrument was based on an existing traffic light-based instrument developed to assess the application of a complex systems perspective in public health-related process evaluations [13]. The eight key concepts included in our instrument are presented in Table 1.

**Table 1:** Key concepts of a complex systems perspective included in the traffic light-based instrument

Concept	Definition
(1) Heterogeneous elements	Distinct system elements that characterize the agents in the system
(2) Levels	A description of the system structure and the level(s) within that structure
(3) Relationships between elements	Connections or interactions between system elements
(4) Presence of feedback loops between elements	Responses between elements that may alter the intervention and its impacts. Can be reinforcing, positive, negative or balancing
(5) Interactions between system levels	Elements at one system level influence elements at other system levels
(6) Adaptation	Adjustments in system behaviour in response to internal and external change
(7) Emergence	Patterns that emerge from the interplay between factors, and system-level behaviour cannot be attributed to its individual parts
(8) Nonlinear dynamics	Inputs into the system do not necessarily result in correspondingly sized effects in the system, and the state of the system changes over time

For each included study, the application of each key concept was assessed using green, yellow and red traffic lights. Green was used when a concept was explicitly incorporated into the model, meaning that the authors described that the concept was applied and how they applied it. Yellow was used when a concept seemed to have been incorporated into the model, but this was not clearly described in the publication. Red was used when a concept was not incorporated into the model.

In addition to the key concepts of a complex systems perspective, several extracted study characteristics related to the applied value of the complex systems models identified in the literature are especially relevant for this analysis. These include the evidence the model (including the model structure and, if relevant, model operationalization) was based on, how the model was presented in the manuscript (i.e. diagram, text or table) and characteristics of the relationships in the model. Relationship characteristics included the direction (going to and from certain model elements), polarity (positive or negative) and magnitude (strength) of the relationships.

Data extraction, including the assessment of the application of key concepts of a complex systems approach, was performed by one reviewer (A.M.). Two reviewers (S.V. and M.P.) validated the data extraction on a total of 20% of the included studies. Any discrepancies were discussed between reviewers (and, if needed, the full research team) until agreement was reached, and insights from these discussions were applied to the data extracted from all studies included in the review.

## Complex systems methods-related results from the systematic scoping review

Table 2 provides an overview of the extent to which the 42 studies included in the systematic scoping review [14–18, 25–61] applied key concepts of a complex systems perspective and characteristics related to the studies’ applied value. As presented in the systematic scoping review [8], key concepts of a complex systems perspective included in our traffic light assessment were applied to varying degrees, and only five studies explicitly applied all key concepts [14–18].

**Table 2:**
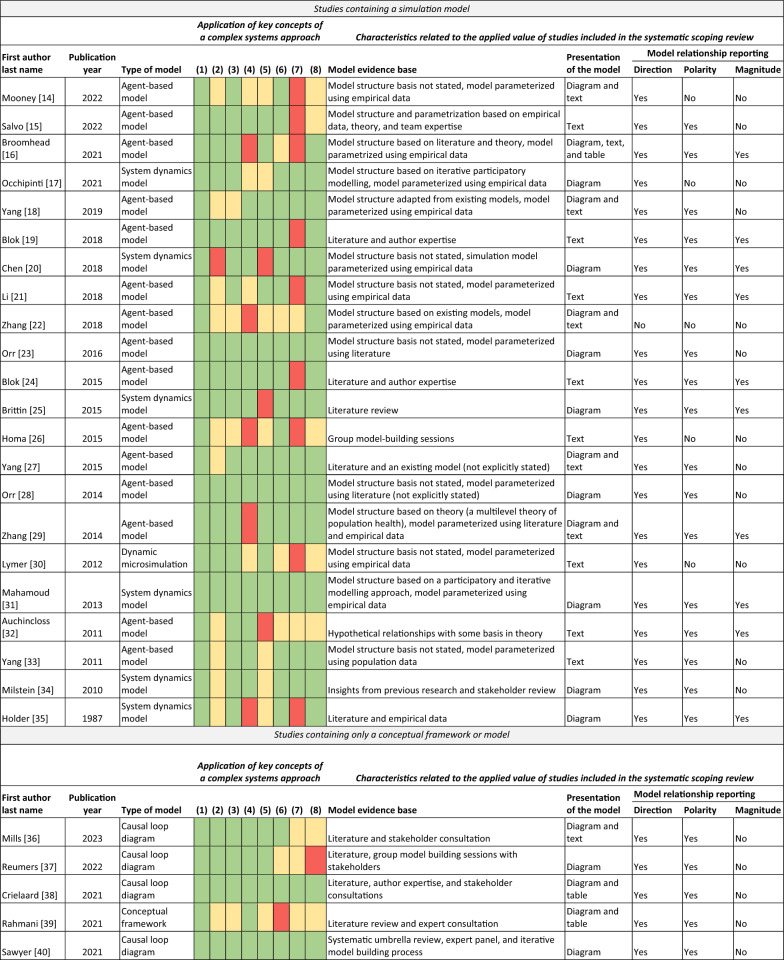

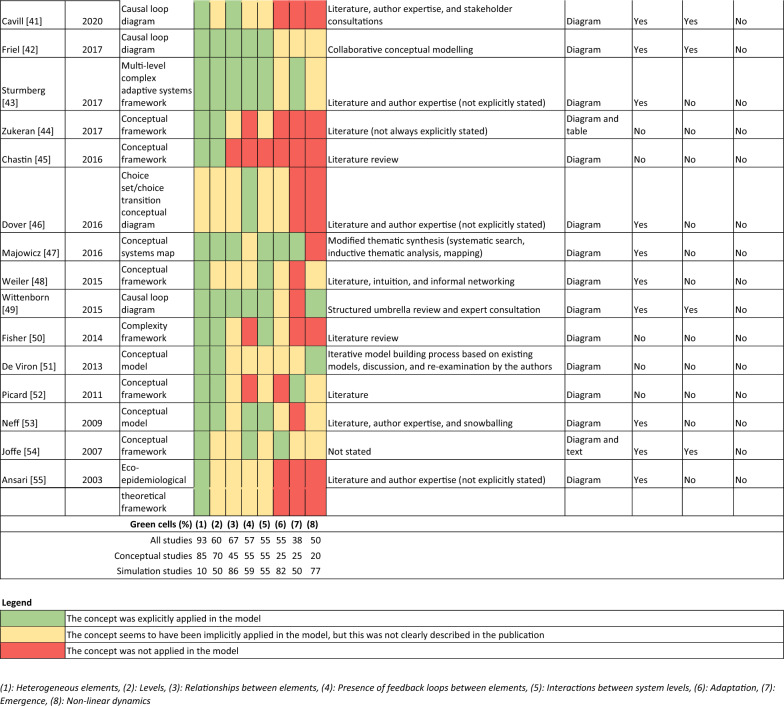
Overview of study characteristics related to complex systems methods

About half (*N* = 23) of the included studies clearly described how the modelled relationships were based on literature, empirical study, iterative model building processes or a combination of these; for the other half, the model evidence base was less clear (Table 2). All studies containing a conceptual model were explicit about the model structure in the sense that they included a diagram of the model structure (e.g. a causal loop diagram), whereas about half of studies containing a simulation model included a diagram. A total of 86% of studies reported the direction of relationships between model elements, 62% reported on polarity and only 24% of studies reported on magnitude. Simulation models contained more detail about the modelled relationships than conceptual models, though conceptual models were not expected to report the magnitude of relationships (direction 95% versus 75%, polarity 77% versus 45%, magnitude 45% versus 0%).

## Further analysis and critical reflection

### Critical appraisal of how a complex systems perspective was applied in literature

One reason that studies containing simulation models may have applied key concepts of a complex systems approach more consistently than studies containing only conceptual models may be the existence of reporting standards for specific types of simulation models. For instance, the overview, design concepts and details protocol (ODD) for agent-based models is widely cited and includes questions about key concepts of complex systems thinking, such as emergence and adaptation [19, 20]. In our analyses, we observed that adaptation was explicitly addressed in 82% of simulation studies (versus 25% of conceptual studies), and emergence was explicitly addressed in 50% of simulation studies (versus 25% of conceptual studies). Still, 50% of simulation studies explicitly considering emergence is not very high, and in fact, the agent-based models included in our review were less likely to consider emergence than other types of simulation models. It seems that, although guidelines such as the ODD protocol are important for the reproducibility and understandability of models, these guidelines may not yet be widely used in agent-based models on socioeconomic inequalities in health.

Studies that applied more key concepts of a complex systems perspective were more likely to report the direction and polarity of the modelled relationships. To investigate this more closely, we estimated Spearman correlations between the percentage of key concepts that were explicitly considered in a model and whether relationship direction, polarity and both direction and polarity were reported on. These additional calculations showed that the percentage of key concepts explicitly considered in a model was positively correlated with reporting the direction (Spearman correlation coefficient of 0.43, *P* value < 0.001), polarity (Spearman correlation coefficient of 0.58, *P* value < 0.001) and combined direction and polarity of relationships (Spearman correlation coefficient of 0.60, *P* value < 0.001). Reporting on direction and polarity increases the applied value of complex systems models. Specifically, knowing whether a model element has a positive or negative influence on health is crucial to understanding model relationships, feedback loops and the functioning of the system as a whole. In our scoping review, reported relationship direction and polarity allowed us to meaningfully interpret shared drivers of socioeconomic inequalities in health and health behaviour and to summarize the existing literature in a causal loop diagram. It could be beneficial to include whether studies report on the direction and polarity of model relationships in assessments of the application of a complex systems perspective in future research. This would go a step further than our traffic light-based assessment of whether (but not how) connections or interactions between system elements were specified.

A key concept of a complex systems perspective that we did not include in our traffic light-based instrument but that may be important to consider is the system paradigm. A complex system’s paradigm is the mindset out of which the system arises or its deepest held belief [21, 22]. The system’s paradigm is the source of its overarching goals, which the system can adapt towards over time [22]. One study included in the scoping review analysed and described the system paradigm: Sawyer and colleagues found that the dynamic system underlying the food environment and its influence on dietary intake in low-income groups was driven by an economic paradigm, with ‘the need for economic prosperity as the system’s deepest held belief’ (15, p.10). This paradigm was elucidated by analysing how the model subsystems were interconnected and related to key dimensions of the food environment. According to the Intervention Level Framework, intervening on the system paradigm has the highest potential for impact on the system, whereas intervening on individual system elements has the lowest potential impact [22]. Despite its purported importance, very few policy recommendations or interventions are aimed at system paradigms, and neither our assessment or other existing assessments of the application of a complex systems perspective included system paradigm as a key concept of a complex systems perspective. This may be because identifying the system’s paradigm is inherently complex. There is no singular strategy for identifying the system’s paradigm, as understanding a system’s paradigm requires thorough and meaningful engagement with the functioning of a complex system. Indeed, building a model of a complex system compels us to view the system as a whole, bringing the system’s goals and paradigm to the surface [21, 22]. As the field develops further, researchers should seek to identify the system paradigm when analysing and assessing complex systems models, as understanding the system paradigm may help bring the most useful and effective policy levers to light.

Finally, including a clear and complete depiction of the model structure was important for assessing the extent to which a complex systems perspective was applied and for being able to extract useful information about the model content. Most diagrams included in simulation studies represented the general model structure, and details about the formalized model were provided in text. Studies containing conceptual models were more likely to include explicit descriptions of the model development process and the evidence the model structure was based on (e.g. literature or stakeholder sessions). On the other hand, studies containing simulation models usually included descriptions of the evidence informing the model parameterization (often empirical data), but the evidence underpinning the chosen model structure itself was often vague or missing. In some cases, text-only descriptions were incomplete, making it challenging to interpret model relationships, and in other cases, diagrams on their own may not be informative enough to understand the model relationships (e.g. if relationship polarity is not indicated in the diagram). Diez Roux emphasized the importance of describing the model structure for conceptual and simulation studies alike: ‘Any systems approach must begin with the development of what has been referred to as a mental model’ (1, p.1631). In the process of extracting model relationships from the included studies, we experienced that it was easier to understand the model if both a diagram and some text describing important model dynamics was available.

### Call for reporting guidelines

Based on this critical appraisal of how conceptual and simulation-based complex systems approaches were applied in the literature on socioeconomic inequalities in health and health behaviour, we call for the development of reporting guidelines for studies that aim to apply a complex systems perspective. Guidelines on reporting standards for a broad range of complex systems models would benefit researchers, those developing models and those interpreting (or aiming to build upon) study findings alike. These guidelines could focus on making the authors’ approach and the model structure understood by readers. In this way, the guidelines would complement rather than replace existing guidelines, such as the ODD protocol [19, 20] or the recently published guidance on the use of complex systems models for economic evaluations of public health interventions [23]. These existing guidelines are more focused on technical aspects of simulation modelling or the development of simulation models for specific purposes (e.g. economic evaluation). In Box 1, we propose some initial recommendations for researchers aiming to apply a complex systems perspective to understanding socioeconomic inequalities in health and beyond, informed by the analysis presented in this manuscript. These suggestions are far from exhaustive and should be expanded on and formalized as comprehensive reporting guidelines. Adherence to such guidelines would likely improve the quality, understandability and applied value of future complex systems models for understanding and tackling socioeconomic inequalities in health.

Box 1: recommendations for researchers aiming to apply a complex systems perspective

1. Explicitly describe how a complex systems perspective was incorporated in the model
  Explicitly describing which concepts of a complex systems perspective were incorporated into the presented model and which ones were not applied and why, will make the alignment between the study aim and the applied method clearer. Below is a list of characteristics of a complex systems perspective that could be described:
  - Heterogeneous elements
  - Levels
  - Relationships between elements, including the direction and polarity of the relationships
  - Presence of feedback loops between elements
  - Interactions between system levels
  - Adaptation
  - Emergence
  - Nonlinear dynamics
  - System paradigm

2. Describe the process of developing the conceptual model and the evidence base underlying the model relationships
  Many different approaches exist for developing conceptual complex systems models, including group model building sessions, literature review, expert knowledge or any combination of these [24]. Knowing more about the development process is essential for the reader to be able to understand the context the model was built in (i.e. what are the perspectives and positionalities of the model developers?) and to assess the validity of the model. This recommendation applies to conceptual and simulation studies alike, as, ideally, the structure of a simulation model is based on a conceptual model

3. Include a diagram depicting the model structure in its entirety
  The inclusion of a complete and informative diagram, such as a causal loop diagram, ensures that readers are able to understand the full model structure. Text-only descriptions can leave gaps in understanding, making it challenging to interpret model relationships and making model outcomes less reliable to the reader. Textual descriptions of the model structure can, however, complement a diagram, especially when authors wish to provide information on the relative importance or magnitude of certain model elements or relationships

## Conclusions

In this short manuscript, we analysed how complex systems methods were used and reported on in existing studies on socioeconomic inequalities in health and health behaviour, in which authors reported applying a complex systems perspective. While key concepts of a complex systems perspective were applied to varying degrees, we found that more thorough reporting on how a complex systems perspective was applied increased the understandability and applied value of models. Specifically, describing how key concepts of a complex systems perspective were applied, providing details about model relationships and presenting a clear justification for and depiction of the full model structure all increased the ability to understand the functioning of complex systems models. While we present reporting-related recommendations for researchers based on our analysis, we emphasize the need for the development of comprehensive reporting guidelines to increase the transparency and applied value of complex systems models on topics related to socioeconomic inequalities in health, health behaviour and beyond.

## Data Availability

The full search strings and key study and model characteristics are available in the manuscript reporting the content-related findings of the systematic scoping review. Findings from the assessment of the assessment of the application of a complex systems perspective and from the assessment of the evidence base are available in Table 2.

## Abbreviations

OECD: Organization for Economic Co-operation and Development
ODD protocol: Overview, Design concepts, Details protocol

## References

[1] Diez Roux AV. Complex systems thinking and current impasses in health disparities research. Am J Public Health. 2011;101:1627–34. 21778505 10.2105/AJPH.2011.300149 PMC3154209

[2] Wilderink L, Bakker I, Schuit AJ, Seidell JC, Pop IA, Renders CM. A theoretical perspective on why socioeconomic health inequalities are persistent: building the case for an effective approach. IJERPH. 2022;19(14):8384. 35886234 10.3390/ijerph19148384 PMC9317352

[3] Rutter H, Savona N, Glonti K, Bibby J, Cummins S, Finegood DT, et al. The need for a complex systems model of evidence for public health. Lancet. 2017;390(10112):2602–4. 28622953 10.1016/S0140-6736(17)31267-9

[4] Carey G, Malbon E, Carey N, Joyce A, Crammond B, Carey A. Systems science and systems thinking for public health: a systematic review of the field. BMJ Open. 2015;5(12): e009002. 26719314 10.1136/bmjopen-2015-009002 PMC4710830

[5] Jayasinghe S. Social determinants of health inequalities: towards a theoretical perspective using systems science. Int J Equity Health. 2015. 10.1186/s12939-015-0205-8. 26303914 10.1186/s12939-015-0205-8 PMC4549102

[6] Kaplan GA, Diez Roux AV, Simon CP, Galea S. Growing inequality: bridging complex systems, population health and health disparities. Washington, DC: Westphalia Press; 2017. p. 332.

[7] Salway S, Green J. Towards a critical complex systems approach to public health. Crit Public Health. 2017;27(5):523–4.

[8] Mudd AL, Bal M, Verra SE, Poelman MP, de Wit J, Kamphuis CBM. The current state of complex systems research on socioeconomic inequalities in health and health behavior—a systematic scoping review. Int J Behav Nutrit Phys Act. 2024. 10.1186/s12966-024-01562-1. 10.1186/s12966-024-01562-1 PMC10845451 38317165

[9] OECD. Country classification 2022—as of 3 August 2022. OECD. 2022. https://www.oecd.org/trade/topics/export-credits/documents/2022-cty-class-en-(valid-from-03-08-2022).pdf. Accessed 12 Apr 2023.

[10] Rutter H, Cavill N, Bauman A, Bull F. Systems approaches to global and national physical activity plans. Bull World Health Organ. 2019;97(2):162–5. 30728623 10.2471/BLT.18.220533 PMC6357559

[11] Carmichael T, Hadžikadić M. The fundamentals of complex adaptive systems. In: Carmichael T, Collins AJ, Hadžikadić M, editors. complex adaptive systems. Cham: Springer; 2019.

[12] Holden LM. Complex adaptive systems: concept analysis. J Adv Nurs. 2005;52(6):651–7. 16313378 10.1111/j.1365-2648.2005.03638.x

[13] McGill E, Marks D, Er V, Penney T, Petticrew M, Egan M. Qualitative process evaluation from a complex systems perspective: a systematic review and framework for public health evaluators. PLoS Med. 2020;17(11):e1003368. 33137099 10.1371/journal.pmed.1003368 PMC7605618

[14] Mooney SJ, Shev AB, Keyes KM, Tracy M, Cerdá M. G-computation and agent-based modeling for social epidemiology: can population interventions prevent posttraumatic stress disorder? Am J Epidemiol. 2022;191(1):188–97. 34409437 10.1093/aje/kwab219 PMC8897987

[15] Salvo D, Lemoine P, Janda KM, Ranjit N, Nielsen A, Van Den Berg A. Exploring the impact of policies to improve geographic and economic access to vegetables among low-income, predominantly Latino urban residents: an agent-based model. Nutrients. 2022;14(3):646. 35277005 10.3390/nu14030646 PMC8839639

[16] Broomhead T, Ballas D, Baker SR. Neighbourhoods and oral health: agent-based modelling of tooth decay. Health Place. 2021;71: 102657. 34543838 10.1016/j.healthplace.2021.102657

[17] Occhipinti JA, Skinner A, Iorfino F, Lawson K, Sturgess J, Burgess W, et al. Reducing youth suicide: systems modelling and simulation to guide targeted investments across the determinants. BMC Med. 2021;19(1):61. 33706764 10.1186/s12916-021-01935-4 PMC7952221

[18] Yang Y, Langellier BA, Stankov I, Purtle J, Nelson KL, Diez Roux AV. Examining the possible impact of daily transport on depression among older adults using an agent-based model. Aging Ment Health. 2019;23(6):743–51. 29543502 10.1080/13607863.2018.1450832

[19] Blok DJ, Van Lenthe FJ, De Vlas SJ. The impact of individual and environmental interventions on income inequalities in sports participation: explorations with an agent-based model. Int J Behav Nutr Phys Act. 2018;15(1):107. 30382862 10.1186/s12966-018-0740-y PMC6211418

[20] Chen HJ, Xue H, Liu S, Huang TTK, Wang YC, Wang Y. Obesity trend in the United States and economic intervention options to change it: a simulation study linking ecological epidemiology and system dynamics modeling. Public Health. 2018;161:20–8. 29857248 10.1016/j.puhe.2018.01.013 PMC6953626

[21] Li Y, Zhang D, Thapa JR, Madondo K, Yi S, Fisher E, et al. Assessing the role of access and price on the consumption of fruits and vegetables across New York City using agent-based modeling. Prev Med. 2018;106:73–8. 28987339 10.1016/j.ypmed.2017.10.014 PMC12056753

[22] Zhang Q, Northridge ME, Jin Z, Metcalf SS. Modeling accessibility of screening and treatment facilities for older adults using transportation networks. Appl Geogr. 2018;93:64–75. 29556112 10.1016/j.apgeog.2018.02.013 PMC5856470

[23] Orr MG, Kaplan GA, Galea S. Neighbourhood food, physical activity, and educational environments and black/white disparities in obesity: a complex systems simulation analysis. J Epidemiol Commun Health. 2016;70(9):862–7. 10.1136/jech-2015-205621 27083491

[24] Blok DJ, De Vlas SJ, Bakker R, Van Lenthe FJ. Reducing income inequalities in food consumption. Am J Prev Med. 2015;49(4):605–13. 26232897 10.1016/j.amepre.2015.03.042

[25] Brittin J, Araz OM, Nam Y, Huang TK. A system dynamics model to simulate sustainable interventions on chronic disease outcomes in an urban community. J Simulat. 2015;9(2):140–55.

[26] Homa L, Rose J, Hovmand PS, Cherng ST, Riolo RL, Kraus A, et al. A participatory model of the paradox of primary care. Ann Family Med. 2015;13(5):456–65. 10.1370/afm.1841 PMC4569454 26371267

[27] Yang Y, Auchincloss AH, Rodriguez DA, Brown DG, Riolo R, Diez-Roux AV. Modeling spatial segregation and travel cost influences on utilitarian walking: towards policy intervention. Comput Environ Urban Syst. 2015;51:59–69. 25733776 10.1016/j.compenvurbsys.2015.01.007 PMC4342617

[28] Orr MG, Galea S, Riddle M, Kaplan GA. Reducing racial disparities in obesity: simulating the effects of improved education and social network influence on diet behavior. Ann Epidemiol. 2014;24(8):563–9. 25084700 10.1016/j.annepidem.2014.05.012

[29] Zhang D, Giabbanelli PJ, Arah OA, Zimmerman FJ. Impact of different policies on unhealthy dietary behaviors in an urban adult population: an agent-based simulation model. Am J Public Health. 2014;104(7):1217–22. 24832414 10.2105/AJPH.2014.301934 PMC4056222

[30] Lymer S, Brown L. Developing a dynamic microsimulation model of the Australian health system: a means to explore impacts of obesity over the next 50 years. Epidemiol Res Int. 2012;2012:1–13.

[31] Mahamoud A, Roche B, Homer J. Modelling the social determinants of health and simulating short-term and long-term intervention impacts for the city of Toronto, Canada. Soc Sci Med. 2013;93:247–55. 23123169 10.1016/j.socscimed.2012.06.036

[32] Auchincloss A, Riolo RL, Brown DG, Cook J, Diez Roux AV. An agent-based model of income inequalities in diet in the context of residential segregation. Am J Prev Med. 2011;40(3):303–11. 21335261 10.1016/j.amepre.2010.10.033 PMC3625685

[33] Yang Y, Diez Roux AV, Auchincloss AH, Rodriguez DA, Brown DG. A spatial agent-based model for the simulation of adults’ daily walking within a city. Am J Prev Med. 2011;40(3):353–61. 21335269 10.1016/j.amepre.2010.11.017 PMC3306662

[34] Milstein B, Homer J, Hirsch G. Analyzing national health reform strategies with a dynamic simulation model. Am J Public Health. 2010;100(5):811–9. 20299653 10.2105/AJPH.2009.174490 PMC2853627

[35] Holder HD, Blose JO. Reduction of community alcohol problems: computer simulation experiments in three counties. J Stud Alcohol. 1987;48(2):124–35. 3560948 10.15288/jsa.1987.48.124

[36] Mills SD, Golden SD, O’Leary MC, Logan P, Hassmiller LK. Using systems science to advance health equity in tobacco control: a causal loop diagram of smoking. Tob Control. 2023;32(3):287–95. 34535509 10.1136/tobaccocontrol-2021-056695 PMC9466654

[37] Reumers L, Bekker M, Hilderink H, Jansen M, Helderman JK, Ruwaard D. Qualitative modelling of social determinants of health using group model building: the case of debt, poverty, and health. Int J Equity Health. 2022;21(1):72. 35590354 10.1186/s12939-022-01676-7 PMC9118602

[38] Crielaard L, Nicolaou M, Sawyer A, Quax R, Stronks K. Understanding the impact of exposure to adverse socioeconomic conditions on chronic stress from a complexity science perspective. BMC Med. 2021;19(1):242. 34635083 10.1186/s12916-021-02106-1 PMC8507143

[39] Rahmani J, Mirzay Razaz J, Kalantari N, Garcia LMT, Shariatpanahi SP, Bawadi H, et al. Dynamic conceptual framework to investigate adoption of healthy diet through agent-based modelling. BFJ. 2021;123(8):2743–55.

[40] Sawyer ADM, Van Lenthe F, Kamphuis CBM, Terragni L, Roos G, Poelman MP, et al. Dynamics of the complex food environment underlying dietary intake in low-income groups: a systems map of associations extracted from a systematic umbrella literature review. Int J Behav Nutr Phys Act. 2021;18(1):96. 34256794 10.1186/s12966-021-01164-1 PMC8276221

[41] Cavill N, Richardson D, Faghy M, Bussell C, Rutter H. Using system mapping to help plan and implement city-wide action to promote physical activity. J Public Health Res. 2020;9(3):jphr.2020.1759. 10.4081/jphr.2020.1759 PMC7459760 32913833

[42] Friel S, Pescud M, Malbon E, Lee A, Carter R, Greenfield J, et al. Using systems science to understand the determinants of inequities in healthy eating. PLoS ONE. 2017;12(11):e0188872. 29190662 10.1371/journal.pone.0188872 PMC5708780

[43] Sturmberg JP, Bennett JM, Martin CM, Picard M. ‘Multimorbidity’ as the manifestation of network disturbances: ‘Multimorbidity’ as network disturbances. J Eval Clin Pract. 2017;23(1):199–208. 27421249 10.1111/jep.12587

[44] Zukeran MS, Ribeiro SML. The importance of nutrition in a conceptual framework of frailty syndrome. Curr Nutr Rep. 2017;6(2):93–101.

[45] Chastin SFM, De Craemer M, Lien N, Bernaards C, Buck C, Oppert JM, et al. The SOS-framework (Systems of Sedentary behaviours): an international transdisciplinary consensus framework for the study of determinants, research priorities and policy on sedentary behaviour across the life course: a DEDIPAC-study. Int J Behav Nutr Phys Act. 2016;13(1):83. 27421750 10.1186/s12966-016-0409-3 PMC4947275

[46] Dover RVH, Lambert EV. “Choice Set” for health behavior in choice-constrained settings to frame research and inform policy: examples of food consumption, obesity and food security. Int J Equity Health. 2016;15(1):48. 26984387 10.1186/s12939-016-0336-6 PMC4793539

[47] Majowicz SE, Meyer SB, Kirkpatrick SI, Graham JL, Shaikh A, Elliott SJ, et al. Food, health, and complexity: towards a conceptual understanding to guide collaborative public health action. BMC Public Health. 2016;16(1):487. 27277001 10.1186/s12889-016-3142-6 PMC4898364

[48] Weiler AM, Hergesheimer C, Brisbois B, Wittman H, Yassi A, Spiegel JM. Food sovereignty, food security and health equity: a meta-narrative mapping exercise. Health Policy Plan. 2015;30(8):1078–92. 25288515 10.1093/heapol/czu109 PMC4559116

[49] Wittenborn AK, Rahmandad H, Rick J, Hosseinichimeh N. Depression as a systemic syndrome: mapping the feedback loops of major depressive disorder. Psychol Med. 2016;46(3):551–62. 26621339 10.1017/S0033291715002044 PMC4737091

[50] Fisher M, Milos D, Baum F, Friel S. Social determinants in an Australian urban region: a ‘complexity’ lens. Health Promot Int. 2014. 10.1093/heapro/dau071. 25107921 10.1093/heapro/dau071

[51] De Viron S, Malats N, Van Der Heyden J, Van Oyen H, Brand A. Environmental and genomic factors as well as interventions influencing smoking cessation: a systematic review of reviews and a proposed working model. Public Health Genomics. 2013;16(4):159–73. 23796797 10.1159/000351453

[52] Picard M, Sabiston CM, McNamara JK. The need for a transdisciplinary, global health framework. J Alternat Complement Med. 2011;17(2):179–84. 10.1089/acm.2010.0149 21309708

[53] Neff RA, Palmer AM, McKenzie SE, Lawrence RS. Food systems and public health disparities. J Hunger Environ Nutrit. 2009;4(3–4):282–314. 23173027 10.1080/19320240903337041 PMC3489131

[54] Joffe M. Health, livelihoods, and nutrition in low-income rural systems. Food Nutr Bull. 2007;28(2_suppl2):227–36. 10.1177/15648265070282S202 17658069

[55] Ansari Z, Carson NJ, Ackland MJ, Vaughan L, Serraglio A. A public health model of the social determinants of health. Soc Prev Med. 2003;48(4):242–51. 10.1007/s00038-003-2052-4 12971112

[56] Achter S, Borit M, Chattoe-Brown E, Siebers PO. RAT-RS: a reporting standard for improving the documentation of data use in agent-based modelling. Int J Soc Res Methodol. 2022;25(4):517–40.

[57] Grimm V, Berger U, Bastiansen F, Eliassen S, Ginot V, Giske J, et al. A standard protocol for describing individual-based and agent-based models. Ecol Model. 2006;198(1–2):115–26.

[58] Meadows DH. Thinking in systems: a primer. London: Earthscan; 2009. p. 218.

[59] Johnston LM, Matteson CL, Finegood DT. Systems science and obesity policy: a novel framework for analyzing and rethinking population-level planning. Am J Public Health. 2014;104(7):1270–8. 24832406 10.2105/AJPH.2014.301884 PMC4056198

[60] Breeze PR, Squires H, Ennis K, Meier P, Hayes K, Lomax N, et al. Guidance on the use of complex systems models for economic evaluations of public health interventions. Health Econ. 2023;32(7):1603–25. 37081811 10.1002/hec.4681 PMC10947434

[61] Baugh Littlejohns L, Hill C, Neudorf C. Diverse approaches to creating and using causal loop diagrams in public health research: recommendations from a scoping review. Public Health Rev. 2021;14(42):1604352. 10.3389/phrs.2021.1604352 PMC8712315 35140995

